# Midgut of the non-hematophagous mosquito *Toxorhynchites theobaldi* (Diptera, Culicidae)

**DOI:** 10.1038/srep15836

**Published:** 2015-10-30

**Authors:** Raquel S. M. Godoy, Kenner M. Fernandes, Gustavo F. Martins

**Affiliations:** 1 Departamento de Biologia Geral—Universidade Federal de Viçosa, 36570-900 Viçosa, Minas Gerais, Brasil

## Abstract

In most mosquito species, the females require a blood-feeding for complete egg development. However, in *Toxorhynchites* mosquitoes, the eggs develop without blood-feeding, and both females and males exclusively feed on sugary diets. The midgut is a well-understood organ in blood-feeding mosquitoes, but little is known about it in non-blood-feeding ones. In the present study, the detailed morphology of the midgut of *Toxorhynchites theobaldi* were investigated using histochemical and ultrastructural methods. The midgut of female and male *T. theobaldi* adults consists of a long, slender anterior midgut (AMG), and a short, dilated posterior midgut (PMG). The AMG is subdivided into AMG1 (short, with folds) and AMG2 (long, without folds). Nerve branches and enteroendocrine cells are present in AMG and PMG, respectively. Compared with the PMG of blood-feeding female mosquitoes, the PMG of *T. theobaldi* is smaller; however, in both mosquitoes, PMG seems be the main region of food digestion and absorption, and protein secretion. The epithelial folds present in the AMG of *T. theobaldi* have not been reported in other mosquitoes; however, the midgut muscle organization and endocrine control of the digestion process are conserved in both *T. theobaldi* and blood-feeding mosquitoes.

The family Culicidae (Diptera) is monophyletic and consists of all mosquito species^1^, including species of the tribe Toxorhynchitini^2^. This tribe includes a single genus, *Toxorhynchites*, comprising approximately 93 species^3^. Unlike most mosquitoes, in *Toxorhynchites*, females are not hematophagous^4^,^5^,^6^. Hence, egg development does not depend on blood supply and, as adults, both males and females feed exclusively on nectar, honey, and other sugary substances^3^,^4^,^7^. Lack of hematophagy is not an exclusive characteristic of *Toxorhynchites* and is shared with other genera (e.g., *Malaya* and *Topomyia*) in the family Culicidae. Among the non-hematophagous mosquitoes, *Toxorhynchites* has a greater number of species and wider geographic distribution^8^, making this genus more representative.

The midgut is the portion of the digestive tract responsible for digestion of food in mosquitoes^9^,^10^. In adult mosquitoes, the midgut has two portions, which differ morphologically and functionally: the anterior midgut (AMG) is mainly associated with sugar digestion and absorption^11^,^12^; and the posterior midgut (PMG), which is an expandable sac whose cells are involved in blood digestion (females exclusively), water regulation, digestive enzyme and peritrophic matrix (PM) component synthesis and secretion, and nutrient absorption^9^,^13^,^14^.

Unlike the PMG, the AMG of adult mosquitoes is well supplied by nerve endings^13^. However, both AMG and PMG are enclosed externally by circular and longitudinal muscles, which assist in food movement and provide structural integrity^10^,^15^. The midgut epithelium is adjacent to the muscle fibers, and is predominantly made up of digestive cells. These cells actively participate in nutrients digestion and absorption, with two typical types of cell membrane specializations: microvilli and basal labyrinth^13^. The other cells not directly involved in digestion include endocrine cells, related to the control of digestive processes through the release of hormones and neuropeptides; and regenerative cells, responsible for the renewal of midgut epithelium^10^,^13^,^16^.

The midgut in blood-feeding female mosquitoes is the site of blood digestion and the gateway for establishment of various human pathogen, including viruses, protozoa, and nematodes^17^,^18^,^19^. This explains why the midgut is one of the most understood organs in mosquitoes. However, there has been little research on the midgut of non-hematophagous mosquitoes, such as *Toxorhynchites*. Therefore, in the present study, the midgut morphological and functional characteristics of female and male *Toxorhynchites theobaldi* were investigated, and the differences between this species and blood-feeding mosquito species were discussed. Additionally, this study will also help in understanding the overall morphophysiology of the Culicidae midgut.

## Results

### General morphology and histology

**Figure 1:**
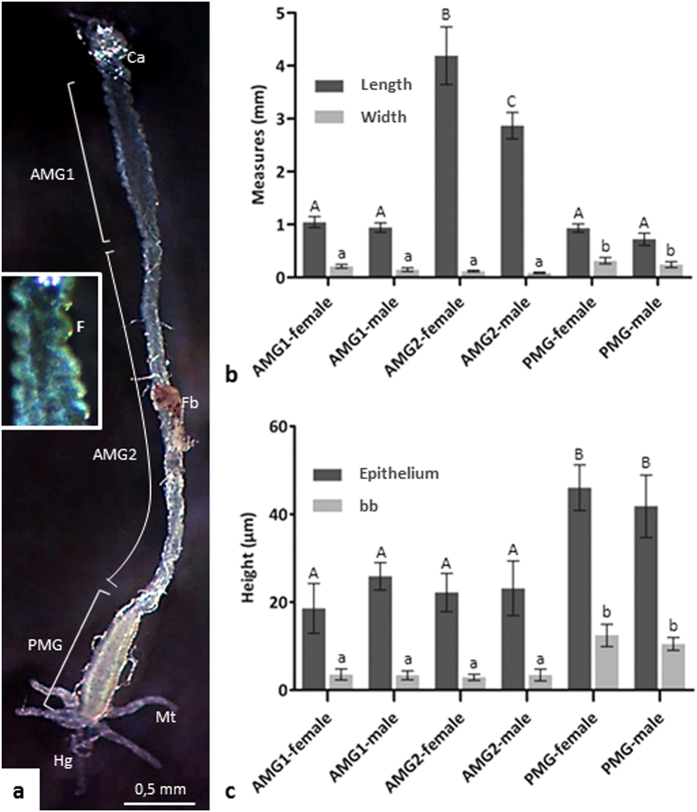
(**a**) Midgut of *Toxorhynchites theobaldi* adult female depicting the anterior midgut (AMG) subdivided in AMG1 (short and with folds) and AMG2 (long and without folds); and a wide and short posterior midgut (PMG). Fb: fat body. Inset: Portion of AMG1 with epithelial folds (F). (**b**) The length and width of the different regions of the midgut are proportional among females and males (p > 0.05). The length of the AMG (AMG1 and AMG2) corresponds to ~84% of the total length of the midgut. (**c**) The heights of the epithelium and the brush border (bb) for each of the three regions of the midgut did not differ between males and females. Bars with the same letter did not differ statistically according to the ANOVA (p < 5%). AMG1: anterior midgut 1; AMG2: anterior midgut 2; PMG: posterior midgut; Ca: cardia; Mt: Malpighian tubule; Hg: hindgut.

The midguts of both female and male *T. theobaldi* consist of a long, slender AMG, and a smaller, dilated PMG. In both females and males, the AMG is divided into two distinct parts: AMG1, with folds on the surface and located in the thorax; and AMG2, without folds and located in abdomen (Fig. 1a and Sup. Fig. a). The total length of the midgut was 6.1 mm in females and 4.5 mm in males, however, length and width of the different regions of the midgut were proportional between females and males. The length of the AMG corresponded to ~84% of the total midgut length. The length of AMG1 corresponded to a quarter of the total length of the AMG. The width of PMG was higher than AMG1 or AMG2 (Fig. 1b).

**Figure 2:**
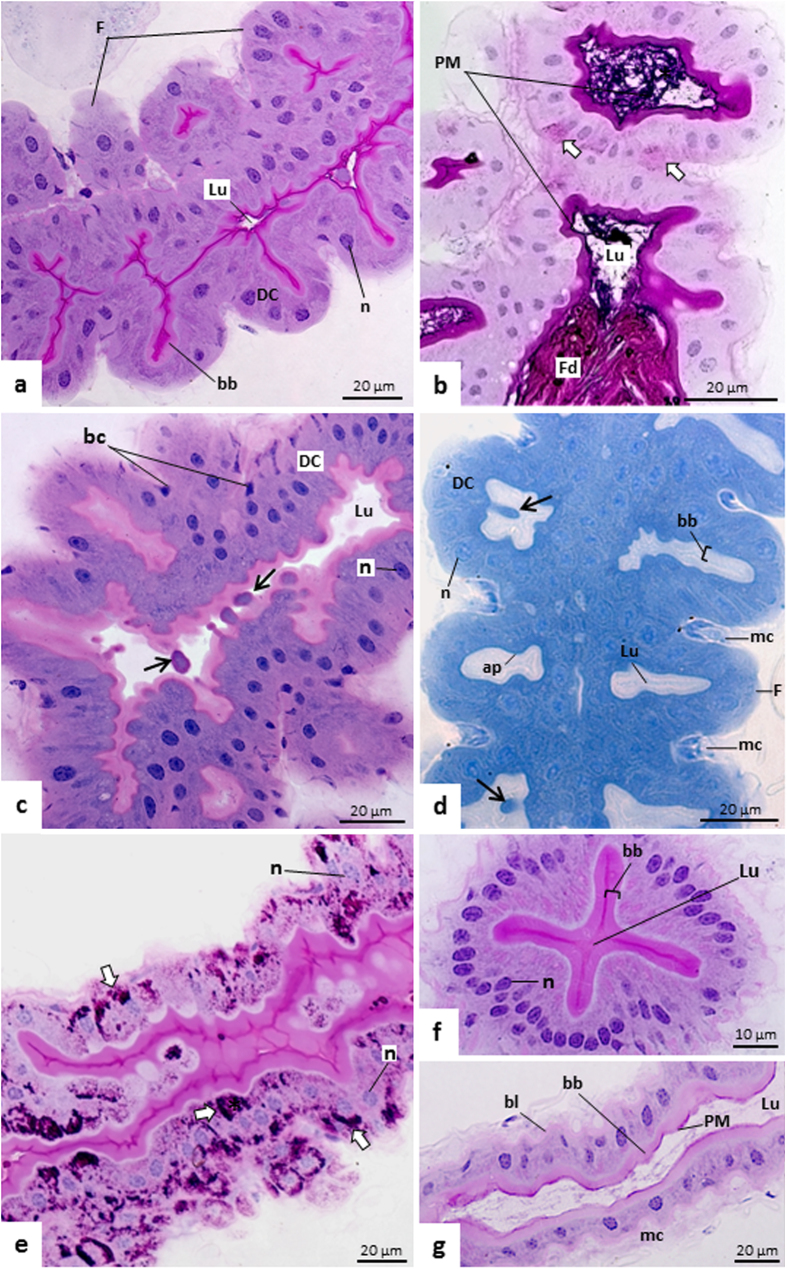
Histological sections of anterior midgut (AMG) of adult *T. theobaldi* stained using periodic acid Schiff (PAS) reaction followed by counterstaining with hematoxylin **(a**,**b**,**e**–**g)**, hematoxylin and eosin **(c)** or bromophenol blue **(d).** (**a)** AMG1 of a male with folds (F), and PAS-positive brush border (bb) n: cell nucleus. (**b**) AMG1 of a female containing food (Fd) and a PAS-positive peritrophic matrix (PM) in the lumen (Lu). Some digestive cells (DC) present PAS-positivity (arrows) in their basal region. (**c**) AMG1 of a female with digestive cells releasing apocrine secretion of acidic character (arrows) into the lumen (Lu). Non-digestive or basal cells (bc) are seen at the basal region of the epithelium. (**d**) AMG1 of a female presenting apical extrusions typical of apocrine secretion, rich in proteins (arrows). The apical portion (ap) of the cells, underneath the brush border, contains a thin layer intensely stained for proteins. Muscle cells (mc) are seen between epithelial folds (F). (**e**) AMG1 of a male showing intense labeling for carbohydrates (arrows) in the digestive cells. (**f**) Cross-section of AMG2 of a female showing cells of different sizes and a X-shaped narrow lumen. (**g**) AMG2 of a male with an undulated basal lamina (bl) where muscle cells (mc) are inserted. The peritrophic matrix (PM) is thin, PAS-positive and is located next to the brush border (bb). Lu: midgut lumen.

**Figure 3:**
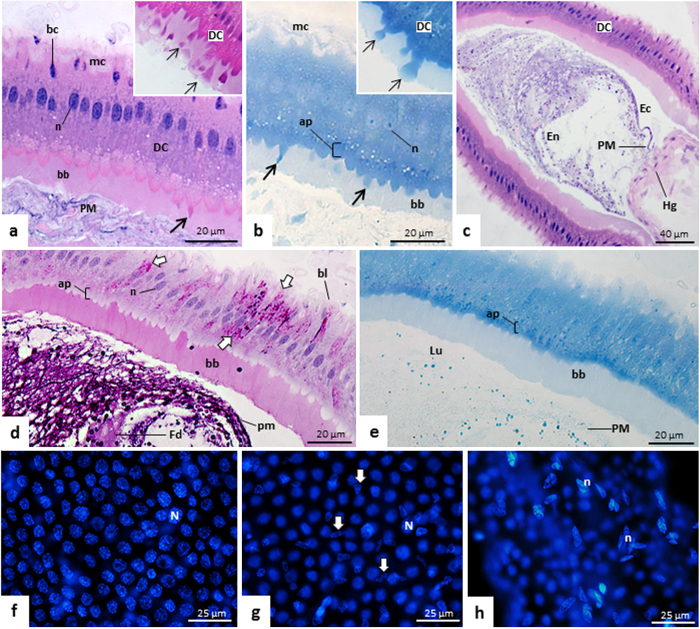
Histological sections of posterior midgut (PMG) of *Toxorhynchites theobaldi* adults stained with hematoxylin and eosin **(a**,**c)**, periodic acid Schiff (PAS) reaction followed by hematoxylin staining **(d)**, and bromophenol blue **(b**,**e)**. **(f**–**h)** Whole mounts of midgut stained with diamidino-2-phenylindole (DAPI). (**a**) Epithelium of a male showing cell apex projections typical of apocrine secretion (arrow) and stained for basic substances. Basal cells (bc) are found throughout the epithelium, near muscle cells (mc). n: nucleus of the digestive cell; PM: peritrophic matrix. (**b**) PMG of a male with the cell projections (arrows). The cell apical region (ap), including the projections, are intensely marked for proteins. bb: brush border; mc: muscle cells. Insets a and b: detailed views of digestive cells (DC) with apical extrusions (arrows) towards midgut lumen, in a process of apocrine secretion. (**c**) Endoperitrophic (En) and ectoperitrophic (Ec) spaces separated by peritrophic matrix (PM) in a female. DC: digestive cells; Hg: hindgut. (**d**) Epithelium of a female with the basal region of the digestive cells with intense staining for carbohydrates (arrows), and with the apical region (ap) negative for carbohydrates. Food (Fd) and peritrophic matrix (PM) are intensely stained, while the apex of the digestive cells (**c**), the brush border (bb) and the basal lamina (bl) are less stained. n: nucleus of the digestive cell. (**e**) Epithelium of a female with a thick apical region (ap) rich in proteins, while the peritrophic matrix (PM) and brush border (bb) are weakly stained. (**f**) Nuclei of digestive cells (N) of PMG of a female. (**g**) Basal cell nuclei (arrows) of the PMG of a female, similar to those observed in AMG1 and 2. The epithelium of the three midgut regions contains non-digestive cells (regenerative or enteroendocrine) with small and irregular nuclei. (**h**) Fusiform nuclei of muscle cells (n) of an AMG1 of a male.

In the three regions of the *T. theobaldi* midgut (AMG1, AMG2, and PMG) there was a single cell layer epithelium with cells displaying brush borders (Figs 2a,g and 3a). The AMG1 epithelium was continuous with the cardia epithelium (proventriculus or the transition between the foregut and midgut) and had many wrinkles or folds (Fig. 2a,d). In AMG2 and PMG, no folds were seen, but undulations occurred in the basal region of the epithelium, where the circular muscles are inserted (Figs 2g and 3a). In AMG1, digestive cells were approximately of the same height (Fig. 2a), unlike AMG2, where cells exhibited different heights, forming a narrow lumen with an “X” shape when cross-sectioned (Fig. 2f). In the PMG, digestive cells were aligned with the nucleus positioned at the same height (Fig. 3a). The height of the epithelium and the thickness of brush border in each of the three-midgut regions were similar between females and males (p > 0.05). Digestive cells in PMG were higher (43.34 μm) and had thicker brush borders (11.31 m) than AMG1 and AMG2, which had similar measurements (22.78 m epithelial height and 3.59 m brush border) (Fig. 1c).

In the three regions of the midgut, digestive cells had elongated or rounded nuclei, and their supranuclear portion was predominantly acidic (Figs 2c and 3a). The subapical cell region, immediately below the brush border, was negative for the PAS reaction, but rich in proteins. This region was thicker or more evident in PMG (Figs 2d and 3d–3e). The brush border on all regions of the midgut was positive for the PAS reaction (Figs 2a,f and 3d). Besides digestive cells, other cells were seen in the basal region of the midgut epithelium (Figs 2c and 3a). These basal cells could correspond to regenerative or enteroendocrine cells. In whole mounts, they had small nuclei with no defined shape, unlike the digestive cells with large, elongated, and regular nuclei (Fig. 3f–g). Externally, the midgut had elongated and large nuclei of muscle cells (Fig. 3h).

The lumen of AMG1 and AMG2 was narrow, sometimes with opposite brush borders very close to each other (Fig. 2a,f), while the PMG lumen was large (Fig. 3c). In AMG1 and PMG, structures protruding from the apical side of the digestive cells towards midgut lumen were visualized, characterizing the process of aprocrine secretion. These structures were positive for proteins (Figs 2d and 3b), and negative for PAS reaction (Supplementary Fig. d,e). In AMG1, these structures were basic, while in PMG, they were acidic (Figs 2c and 3a). This type of cell process was not seen in AMG2.

In midguts where the food bolus is being transferred to the hindgut, digestive cells of AMG and PMG exhibited intense staining for the PAS reaction. This staining was observed in the apical and basal regions, or only in the basal region of cells (Figs 2e and 3d).

**Figure 4:**
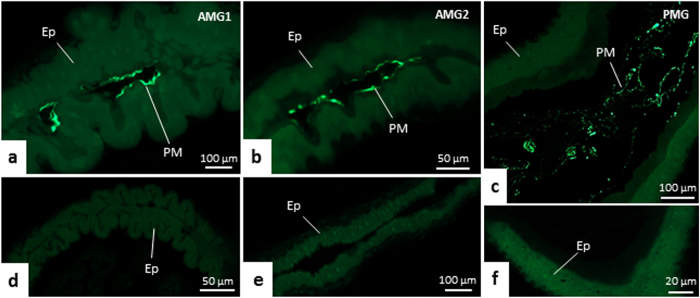
Histological sections of the midgut of adult males of *Toxorhynchites theobaldi* stained with WGA-FITC **(a**–**c)** and negative controls **(d**–**f)**. AMG1: anterior midgut 1; AMG2: anterior midgut 2; PMG: posterior midgut. Ep: midgut epithelium.

A PAS-positive material, corresponding to the PM, was also seen in the lumen of midgut, including inside epithelial folds of AMG1 (Figs 2b,g and 3d). This PM was thin and loosely organized (Figs 2b and 3d). The presence of PM in the lumen of all midgut regions of *T. theobaldi* were confirmed by WGA-FITC staining (Fig. 4a–f). In the AMG1 (Fig. 4a) and AMG2 (Fig. 4b), the labelling was seen just above the brush border. Differently, the labelling in PMG were more diffused or located in the center of the lumen (Fig. 4c).

**Figure 5:**
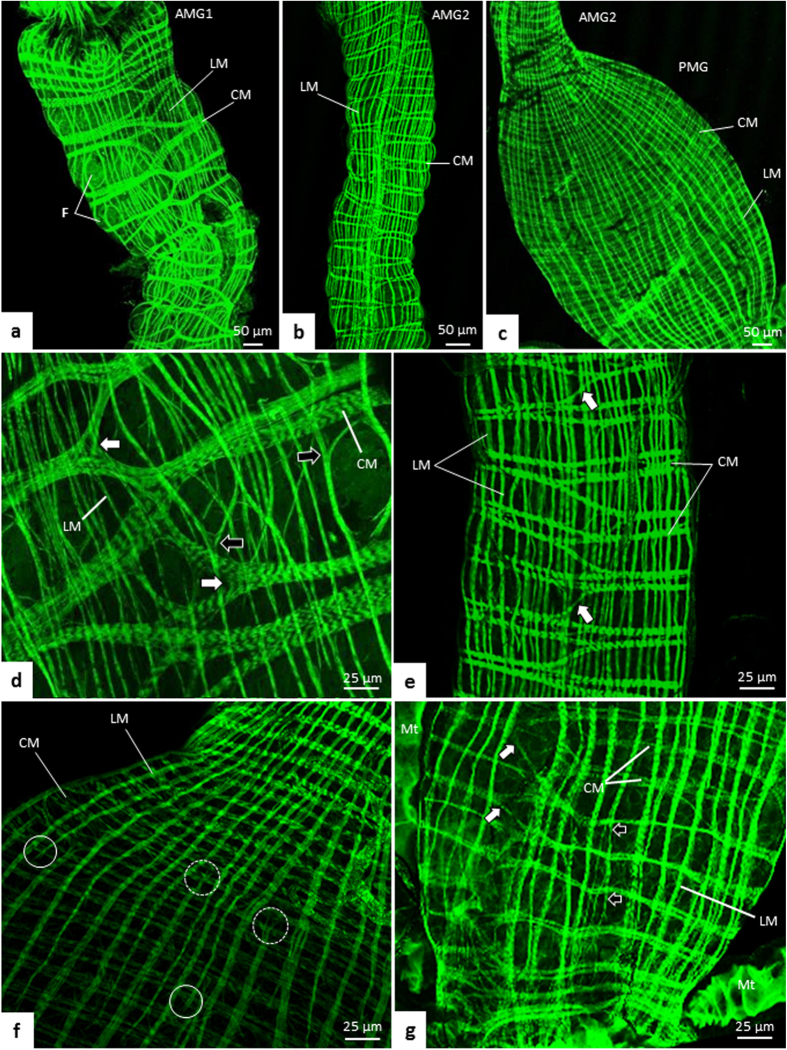
Organization of longitudinal (LM) and circular (CM) muscle bundles of the midgut of *Toxorhynchites theobaldi* adults stained with phalloidin-FITC. (**a**–**c**) anterior midgut 1 (AMG1), anterior midgut 2 (AMG2) and posterior midgut (PMG) of female, respectively. F: epithelial fold. (**d**) Portion of AMG1 of a female with circular muscle bundles, which are thick, bifurcated (white arrow), and interconnected with neighboring bundles. Some ramifications are also seen in the longitudinal muscle bundles (black arrows). (**e**) Portion of AMG2 with circular muscle bundles (CM) forming interconnected rings between neighboring rings through bifurcations (arrows) in a repeated pattern. The longitudinal muscle bundles (LM) are continuous and without ramifications. (**f**) Initial region of the PMG of a female. The longitudinal muscles have some bundles with free ends at the beginning of the PMG. Some of these discontinuous bundles arise from AMG2 (continuous circle) and others from the hindgut (doted circles). (**g**) PMG of a female with circular muscle bundles (CM) with bifurcations (white arrows). Close to the insertion of the Malpighian tubules (Mt), the longitudinal muscles (LM) branch (black arrows).

By phalloidin-FITC labeling (actin marker), muscle bundles were seen in the midgut of *T. theobaldi*, forming a network covering the outer wall of the organ. This labeling revealed the arrangement of circular and longitudinal bundles in the three regions of the midgut (Fig. 5a–c). The longitudinal bundles had similar width in the AMG and were apparently thicker in the PMG, whereas the circular bundles were thicker in AMG1, becoming narrower in the passage between AMG1 and AMG2 (Fig. 5d–f).

The circular bundles were organized orthogonally to the longitudinal ones. Each circular bundle was interconnected to neighbor circular bundles. The interconnection of the circular muscle bundles always occurred at the same position along the midgut length (Fig. 5d,e,g).

The longitudinal bundles were parallel and with few branches, seen only in some muscle bundles of AMG1 and PMG (Fig. 5d,g). The longitudinal bundles were not all continuous from the beginning to the end of the midgut, with some of them terminating early in the PMG, while others originated from the rear end of the PMG, extending the transition of AMG2 to PMG (Fig. 5f).

### Transmission electron microscopy

#### AMG1

**Figure 6:**
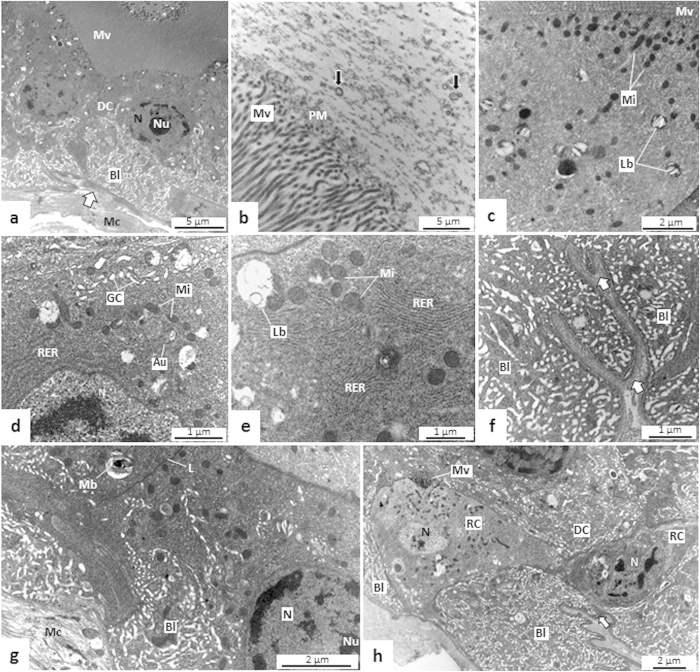
Transmission electron micrographs (TEMs) of anterior midgut 1 (AMG1) of *Toxorhynchites theobaldi* adults. (**a**) Digestive cells (DC) with tall microvilli (Mv) and a well-developed basal labyrinth (Bl) in a male. Mc: muscle cells close to the basal lamina (arrow). (**b**) Peritrophic matrix (PM) with a granular appearance and structures resembling microvesicles (arrows) are seen close to the microvilli (Mv) of a female. (**c**) Apical portion of digestive cell rich in mitochondria (Mi) and lamellar bodies (Lb). Mv: microvilli. (**d**) Golgi apparatus (GC), rough endoplasmic reticulum (RER), mitochondria (Mi) and autophagyc vacuole (Au) on digestive cell of a male. N: nucleus. (**e**) Lamellae of rough endoplasmic reticulum (RER), and lamellar bodies (Lb) in digestive cell of a male. (**f**) Basal labyrinth (Bl) and basal lamina (arrow) with branches in a digestive cell of a male. (**g**) Digestive cell with multilamellar body (Mb) in a male. Bl: basal labyrinth; L: cell limit; Mc: muscle cell; N: nucleus; Nu: nucleolus. (**h**) Two regenerative cells (RC) in the region of the basal labyrinth (Bl) of digestive cells (DC) in a male. One of the regenerative cells (left) is in the differentiation process, with primordial microvilli (Mv) and basal labyrinth (Bl). N: nucleus.

The AMG1 digestive cells had densely-packed microvilli in the apical region and invaginations in the basal region, forming an extensive and sparse labyrinth, which occupied nearly half the cell (Fig. 6a,f). The microvilli were thin, tall, numerous, and contained extracellular material with granular aspect on its ends, corresponding to the PM (Fig. 6b).

The digestive cells of AMG1 had many mitochondria and lamellar bodies in the apex (Fig. 6c). Golgi apparatus, small autophagic vacuoles, lamellar, and multilamellar bodies were also seen (Fig. 6d,e,g). The basal lamina was compact and continuous, and had undulations and depressions (Fig. 6f,h). Muscle cells were located adjacent to the basal lamina (Fig. 6a,g).

**Figure 7:**
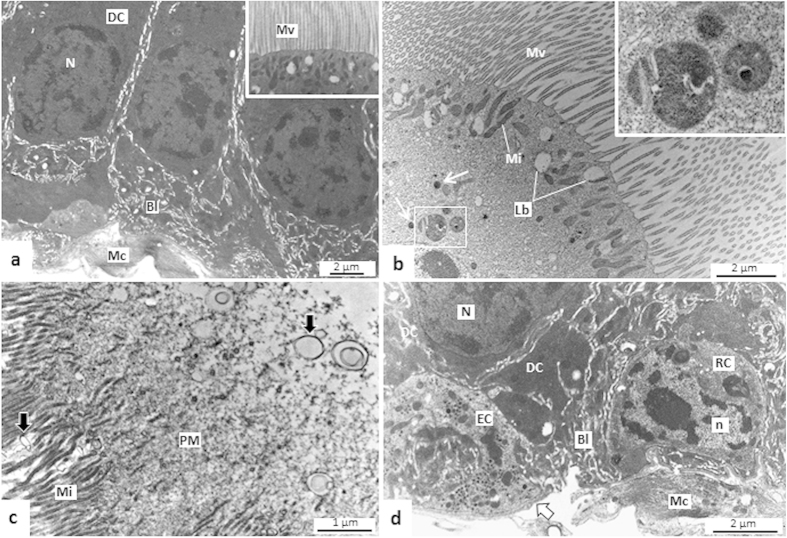
Transmission electron micrographs (TEMs) of the anterior midgut 2 (AMG2) of *Toxorhynchites theobaldi* adults. (**a**) Digestive cells (DC) with thin and densely packed microvilli (Mv, inset), and well-developed basal labyrinth (Bl) in a female. Mc: muscle cell. (**b**) Apical portion of a digestive cell with mitochondria (Mi), lysosome-like structures (arrows), lamellar bodies (Lb), and structures resembling autophagic vacuoles (inset). (**c**) Structures resembling microapocrine vesicles (arrows) close to the microvilli (Mi) of digestive cell in a female. PM: peritrophic matrix. (**d**) Enteroendocrine cell (EC) in contact with the basal lamina (arrow) close to a regenerative cell (RC) in a male. Mc: muscle cell; N and n: nuclei of digestive cell and regenerative cell, respectively.

Regenerative cells were seen in the basal region of the epithelium of AMG1, AMG2, and PMG (Figs 6h and 7d). These small cells had few organelles and extensive lateral expansions, which establish a connection with the neighboring regenerative cells and the basal lamina. Differentiating regenerative cells had emerging microvilli and basal labyrinths (Fig. 6h).

#### AMG2

AMG digestive cells had long and slender microvilli (Fig. 7a, inset and 7b). As well as in AMG1, microvesicle-like structures were seen close to the PM, with single or double layers (Fig. 7c). The digestive cell cytoplasm had autophagic vacuoles (Fig. 7b, inset), electrondense lysosome-like structures, and many mitochondria and lamellar bodies concentrated in the apex (Fig. 7b). The basal labyrinth was extensive, but less developed than in AMG1, and the basal lamina was compact and continuous (Fig. 7d).

**Figure 8:**
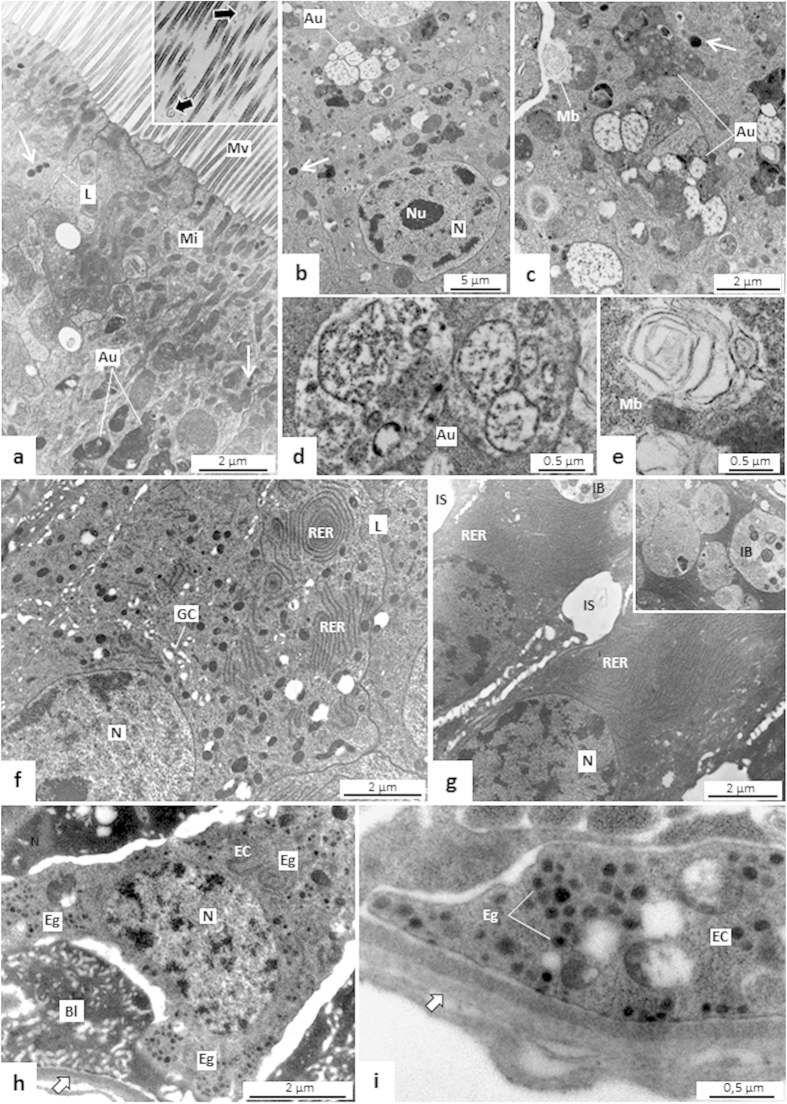
Transmission electron micrographs (TEMs) of the posterior midgut (PMG) of *Toxorhynchites theobaldi* adults. (**a)** Apex of digestive cell with slender and densely clustered microvilli (Mv) in male. Under the microvilli, there are many mitochondria (Mi), autophagic vacuoles (Au) and lysosome-like structures (arrows). Inset: structures resembling microapocrine vesicles (arrows) close to microvilli; L: cell limit. (**b)** Digestive cells with many autophagic vacuoles (Au), lysosome-like structures (arrows) in a female. (**c)** Autophagic vacuoles (Au) in a digestive cell of a female, and a multilamellar body (Mb) released into the intercellular space. (**d**,**e**) Autophagic vacuoles (Au) and multilamellar body (Mb). (**f**) Supranuclear portion of a digestive cell with numerous lamellae of rough endoplasmic reticulum (RER) and Golgi apparatus (GC) in a male. (**g**) Digestive cells filled with rough endoplasmic reticulum (RER) and inclusion bodies (IB, inset). IS: intercellular space; N- nucleus. (**h**) Enteroendocrine cell (EC) with cytoplasm rich in small electrondense granules (Eg) in a female. (**i**) Details of electrondense granules (Eg) of enteroendocrine cell (EC) in a female. Arrow: basal lamina.

Enteroendocrine cells were seen in AMG2, and in PMG. In both regions, these cells had electron-lucent nuclei, few mitochondria, and many small electrondense granules. These cells established contact with the basal lamina through extensive cytoplasmic processes (Figs 7d and 8h,i).

#### PMG

The microvilli of digestive cells of PMG were thin, numerous, and higher than that of AMG and were also associated to microvesicle-like structures (Fig. 8a, inset). Autophagic vacuoles of various sizes, multilamellar bodies, and Golgi apparatus were also found here (Fig. 8b–f). Digestive cells in the PMG were rich in rough endoplasmic reticulum with their concentric lamellae accumulated in the supranuclear region (Fig. 8f–g). Large vesicles, or inclusion bodies, containing eletrondense structures or a granular material (Fig. 8g, inset) were seen in the apex of the digestive cells. Basal labyrinth in PMG was less expressive and the basal lamina was thick and compact in some intervals in comparison to AMG (Fig. 8h,i).

### Scanning electron microscopy

**Figure 9:**
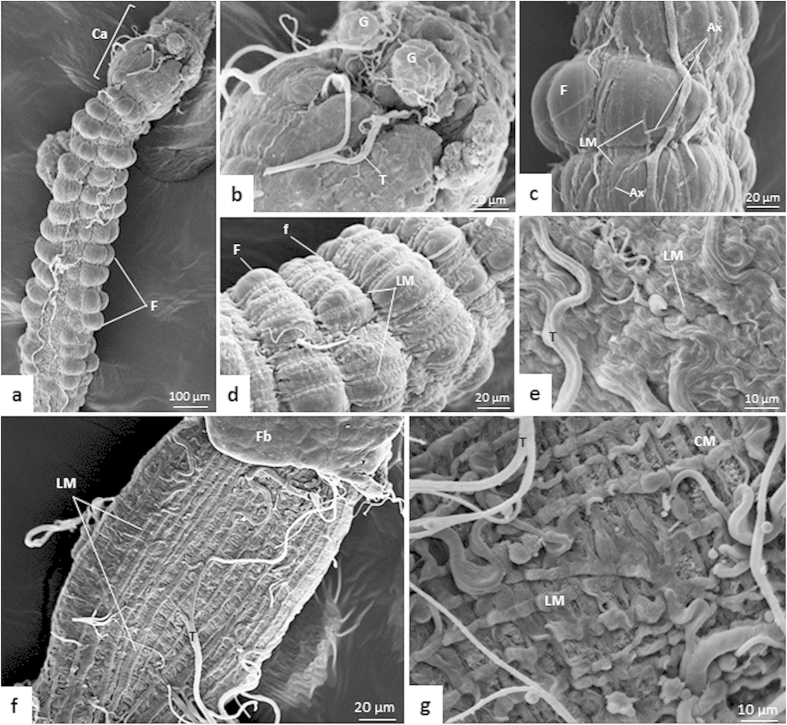
Scanning electron micrographs (SEMs) of the midgut of *Toxorhynchites theobaldi* adults. (**a**) Outer surface of anterior midgut 1 (AMG1) with folds (F) in a female. The cardia (Ca) is positioned between the foregut and midgut. (**b**) Ganglia (G) in AMG1 of a female. T: trachea. (**c**) Longitudinal muscles (LM), and axons (Ax) in the AMG1 of a female. F: epithelial fold. (**d**) AMG1 with folds, showing the furrows (f) and the longitudinal muscles (LM). (**e)** Longitudinal muscles (LM) and tracheoles (T) in anterior midgut 2 (AMG2) of a male. (**f**) PMG with longitudinal muscles (LM) in a female. Fb: fat body. (**g**) Posterior midgut (PMG) depicting the circular muscles (CM) below the longitudinal muscles (LM). T: trachea.

The midgut topography was similar in *T. theobaldi* females and males. AMG1 was continuous with the cardia, a dilated structure that connected the esophagus to AMG (Fig. 9a,b). As seen in the histological sections, the AMG1 epithelium had folds (Fig. 9a,d) that were not seen in AMG2 (Fig. 9e).

Ganglia were located just above the cardia and nerve fibers extended along AMG. Nerves ramified and connected to the longitudinal muscle bundles (Fig. 9c). Tracheoles were seen on the entire surface of the midgut and were most commonly found in AMG2 and PMG (Fig. 9e,f).

In AMG1 and AMG2, only the longitudinal muscle bundles were seen (Fig. 9c–e) under SEM. In AMG1, longitudinal bundles were more widely spaced, but the circular bundles still could not be seen, as they were hidden in the furrows formed between the epithelial folds (Fig. 9d). In AMG2, there were many tracheoles and the longitudinal bundles were very close to each other, hiding the circular bundles (Fig. 9e). In the PMG, the longitudinal bundles were widely spaced, allowing the visualization of circular muscle bundles (Fig. 9f,g).

### Cell proliferation

Cell proliferation was not detected in any of the three regions in the midgut of 5- to 10-day-old adult mosquitoes under experiment conditions. However, in the positive control, corresponding to the midguts of *A. aegypti* (4^th^ larval stage), labeled nuclei were present as expected (Sup. Fig. f).

### FMRFamide-like positive cells

**Figure 10:**
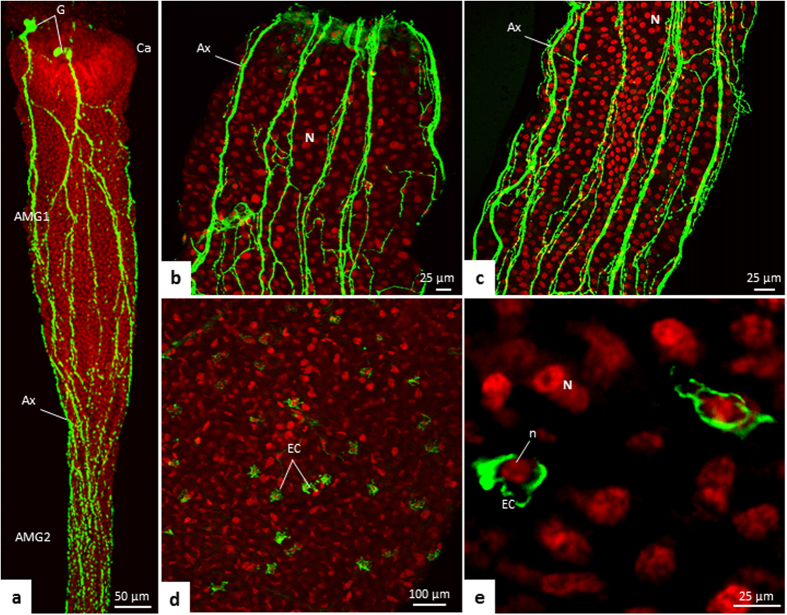
Immunofluorescence for neuropeptide FMRFamide (green) in the midgut of a female *Toxorhynchites theobaldi* adult. The nuclei of the digestive (N) or endocrine (n) cells are marked with TO-PRO-3 Iodide (red). (**a)** Anterior midgut 1 (AMG1) and anterior part of the anterior midgut 2 (AMG2). Ganglia (G) are located on the cardia, and axons (Ax) are seen along the AMG. (**b**,**c)** Axons (green) in AMG1 and AMG2, respectively. (**d**,**e**) FMRFamide enteroendocrine cells (EC) in posterior midgut (PMG).

The anti-FMRFamide antibodies labeled neurons and endocrine cells in *T. theobaldi* midgut. The pattern of this labeling was similar in female and male *T. theobaldi* adults. FMRFamide-like positive ganglions were seen above the cardia and their ramifications were seen overlying more than half of the AMG (Fig. 10a–c).

Enteroendocrine cells (i.e., FMRFamide-like positive cells) were abundant and scattered among the digestive cells of the extreme end of AMG2 (close to PMG) and throughout PMG (Fig. 10d,e). The number of enteroendocrine cells was similar in males and females (p = 0.842), with approximately 99 cells per midgut.

## Discussion

The general morphology of the midgut in female and male *T. theobaldi* resembled that of the midgut of male mosquitoes whose females are hematophagous. In this regard, similar to these males, the AMG of *T. theobaldi* was thin and long, while the PMG was enlarged and reduced in size. Different of this, in blood-feeding female mosquitoes, the AMG is short and the PMG is expanded (Sup. Figs. b and c)^13^,^20^,^21^,^22^,^23^.

The AMG of *T. theobaldi* was subdivided into two morphologically distinct regions: AMG1 and AMG2. In other Culicidae (both females and males) this subdivision is not evident (or absent), and the AMG is slender and without folds, similar to AMG2 of *T. theobaldi*. By being wider than AMG2 and containing folds, AMG1 seems to function as a first site of food digestion. The presence of folds increase the contact surface between the food and the digestive epithelium, and probably reduce the speed in which nutrients pass through the lumen, facilitating the digestion and absorption^24^.

The epithelium characteristics of the three midgut regions of *T. theobaldi* were compatible with the secretory, digestive, absorptive, and nutrient transport functions as reported elsewhere^13^,^25^. Both AMG1 and PMG seem to be more involved in enzyme secretion and nutrient absorption compared with AMG2. These two regions presented apocrine secretion of proteins and intense labeling for carbohydrates, especially in the basal portion of the digestive cells. However, the acidic character of apocrine secretion in AMG1 versus the basic character of this secretion in PMG indicates that the secreted proteins are probably different in the two regions.

The AMG1 and PMG digestive cells of *T. theobaldi* showed greater carbohydrates accumulation when food was being transferred to the hindgut. The carbohydrates accumulation, such as glycogen, is common in insect digestive cells during absorption activity^10^,^26^, and in the PMG digestive cells of larval and adult mosquitoes fed with sugar or blood^27^,^28^,^29^. These carbohydrate deposits seen in PMG seem to accumulate because of the digestion process, functioning as energy reserves, or facilitating the subsequent absorption of more carbohydrates^30^.

Apocrine secretions are typically released during the digestive process of insects, and it is speculated that this is also related to regions that perform nutrients absorption^31^. Corroborating this, AMG1 and PMG are apparently more involved with the carbohydrate absorption, and are the regions where apocrine secretion occurred.

Another possibly secretory mechanism present in the midgut of *T. theobaldi* is the microapocrine secretion. The small single and double membrane structures seen across the midgut lumen of *T. theobaldi* resemble microapocrine secreted vesicles found in the midgut lumen of various insects^31^. Enzymes, such as amylase, and various peritrofins are released into the midgut lumen by this secretory mechanism^32^. The existence of this type of secretory mechanism is something that needed to be clarified in adults of non-hematophagous mosquitoes.

The abundant rough endoplasmic reticulum (RER) lamellae in the PMG are also found in the PMG of hematophagous females when a blood meal is acquired. The marked presence of these organelles occurs in cells that are specialized in protein secretion^9^,^13^,^29^. Accordingly, it is possible to infer that the bloodmeal in blood-feeding female mosquitoes, and the sugar meal in *T. theobaldi* stimulate intense activity of protein secretion in PMG digestive cells. In addition to the PMG, the AMG1 also had many RER lamellae in the digestive cells, which is probably related to the apocrine secretion of proteins as demonstrated by histochemistry with bromophenol blue.

Autophagic vacuoles were seen in the digestive cells of all regions of *T. theobaldi* midgut, being larger in size and quantity in PMG. These vacuoles are related to the recycling of membranes that occurs due to endo- and exocytosis during digestion^29^. Large inclusion bodies were also seen in the digestive cells of *T. theobaldi*, similar to those observed in the PMG of blood-feeding mosquitoes post bloodmeal^16^,^29^. The function of these inclusion bodies is unknown, but it has been proposed for recycling membranes, along with the autophagic vacuoles^16^. By containing a large amount of autophagic vacuoles and inclusion bodies compared with AMG, PMG digestive cells may be more involved in vesicular transport than AMG digestive cells.

Lamellar bodies were also abundantly found in digestive cells throughout the *T. theobaldi* midgut. These structures are composed primarily of lipids and proteins, and their biogenesis involves endocytic and/or autophagic pathways^33^. In some vertebrate digestive epithelia, lamellar bodies are secreted to protect cell membranes from digestive enzymes and the abrasion of food flow^34^. However, the function of these organelles in mosquitoes is unknown.

The PAS reaction and WGA staining^35^ confirmed the presence of a thin PM throughout the midgut lumen in *T. theobaldi*. This PM had a granular/loose appearance under TEM. In hematophagous mosquitoes (adults), the PM is of type I^35^,^36^, however, for the non-hematophagous *T. theobaldi*, the classification of PM (if it is type I or II) is still unclear. In comparison with the PM of *T. theobaldi*, the PM of hematophagous mosquitoes is thicker, more compact, and is found only in the PMG^29^,^37^,^38^,^39^. The presence of PM in *T. theobaldi* midgut indicates that this structure is not only related to blood digestion in Culicidae, but also plays a role in sugar digestion of non-hematophagous species of this family. In the latter case, PM would be important to protect the midgut cells against microorganisms, which are found abundantly in sugar-based foods such as nectar and honey^40^.

Cell divisions could not be identified in the midgut of 5- to 10-day-old *T. theobaldi* adults through antibodies against PH3. The absence of cell division was also reported in adults of *A. aegypti* (with the exception of newly emerged *A. aegypti*) and *Culex quinquefasciatus*^41^,^42^. The dynamics of stem cells division and differentiation in *T. theolbadi* midgut need more investigations considering different ages and digestive phases.

The circular muscle bundles of *T. theobaldi* midgut are interconnected with adjacent circular bundles. This characteristic also occurs in adult female *A. aegypti* and *Anopheles gambiae*^15^. In these two hematophagous species, as well as in *T. theobaldi*, not all PMG longitudinal bundles are continuous. Some bundles emanate from AMG, ending close to the AMG/PMG transition. Additionally, other longitudinal bundles extend from the hindgut towards the PMG surface^15^. These structural similarities between the midgut muscles of *T. theobaldi* and blood-feeding females indicate the existence of a common organization of muscle fibers in mosquitoes, even with markedly different feeding habits.

The ganglia associated with the cardia are part of the stomatogastric nervous system of insects^43^,^44^. FMRFamide-like immunoreactive neurons in *T. theobaldi* innervate only the anterior portion of the midgut, as in other mosquitoes previously studied^43^. Neuropeptides, such as FMRFamide-like peptides (FLPs), are secreted by neurons and endocrine cells, and supposedly these peptides act in the control of the digestive process^43^,^45^,^46^. The physiological roles of these peptides in mosquito midguts are unknown, but in some insects the FLPs look like to be involved in the control of gut motility and secretion of digestive enzymes^47^,^48^,^49^,^50^.

FMRF-like immunoreactive (enteroendocrine) cells are found in the PMG and in the final portion of AMG2 of *T. theobaldi* adults, while in *A. aegypti* adults, these cells are only seen in the PMG^22^,^42^,^43^,^47^. Despite this minor difference in the location of enterodocrine cells, the neuroendocrine control of the digestive process based on FMRFamide-like neuropeptides, in both blood-feeding and in non-blood-feeding mosquitoes, seems to be performed by the ramifications of the stomatogastric nervous system in AMG, and the endocrine cells in PMG. The enteroendocrine cells of *T. theobaldi* present abundant small, electrondense secretory granules, as well as the enteroendocrine cells described elsewhere^16^. Thus, the presence of these granules is another feature conserved among adult mosquitoes with different diets.

The presence of a midgut with a long AMG and reduced PMG in *T. theobaldi* adults is likely an adaptation to sugar-rich diets. Other adaptations to this diet include modifications of the stimuli perception^51^, as in the morphophysiology of salivary glands^52^. Compared to blood-feeding females, *Toxorhynchites amboinensis* females lost some types of chemoreceptors in the palps and antennae, such as a putative ionotropic receptor and various odorant receptors. This simplification of the chemoreceptive repertoire probably resulted from the loss of the blood-feeding habit by *Toxorhynchites*^51^. Additionally, *Toxorhynchites splendens* salivary glands lack secretory cavities and are morphologically and biochemically similar between male and female, unlike in blood-feeding species, where these glands are sexually dimorphic, and females synthesize a range of proteins related to blood-feeding habits^52^.

Through our observations, we conclude that the midgut features that are similar among *T. theobaldi* and hematophagous females include: (1) PMG is rich in specialized organelles for protein secretion; (2) the muscular organization in PMG involves sharing of muscle fibers between neighboring muscle bundles; (3) longitudinal muscle bundles are not continuous along the organ; (4) regenerative cell divisions were not detected in aged adults; and (5) FMRF-like immunoreactive cells, including nervous cells and endocrine cells, are located in AMG and in PMG, respectively. However, the differences between the midguts of *T. theobaldi* and hematophagous females include: (1) AMG in *T. theobaldi* is subdivided into two anatomically distinct regions, AMG1 and AMG2, while in hematophagous females this subdivision is not evident; (2) AMG is very long and PMG is small in *T. theobaldi* when compared with hematophagous females; (3) PM is very thin and is observed in the whole midgut of *T. theobaldi*, but thick, compact, and is synthesized only after a bloodmeal in hematophagous females.

Our results indicate that the morphophysiology of the midgut of the autogenous and sugar-feeding mosquito *T. theobaldi* is similar in both males and females, unlike blood-feeding mosquito species, where sexual dimorphism is evident. This similarity can be ascribed to both female and male *T. theobaldi* having the same feeding behavior. Information on protein synthesis by the midgut of these mosquitoes can unravel the differences in morphology and physiology between the midguts of *T. theobaldi* and hematophagous mosquitoes; however, to date, nothing is known regarding this in the genus *Toxorhynchites*. Studies focusing on the enzymatic activity and proteomics of the midgut in *Toxorhynchites* species are the next steps to improve the understanding of midgut physiology in these insects, providing new insights into the evolutionary adaptations of the family Culicidae related to a sugar-based diet.

## Material and Methods

### Mosquitoes

Immature specimens of *T. theobaldi* (larvae of different stages and pupae) were collected from Mata do Paraíso (20°45′14″S, 42°52′55″W), Atlantic Forest region of Viçosa, Minas Gerais, Brazil. Larvae and pupae were collected in black plastic buckets (5 L) containing rainwater at ground level, near tree trunks. The specimens were transferred to the insectary of the Departamento de Biologia Geral/UFV, where the larvae were reared individually in transparent glass vials (200 mL) and fed daily with *Aedes aegypti* larvae at different stages.

Pupae were transferred to plastic pots containing dechlorinated tap water and kept in cages until the emergence of adults. Adults were fed ad libitum with 10% sucrose solution, and dissected 5–10 d after emergence. The insects were kept at a temperature of 26 ± 2 °C and humidity 60–70%.

### Whole mounting and histology

Twenty midguts (10 males and 10 females) were dissected in phosphate buffered saline—PBS pH 7.6 (0.1 M NaCl, 20 mM KH2PO4, and 20 mM Na_2_HP_4_) and fixed in 2.5% glutaraldehyde solution (sucrose/cacodylate buffer 0.1 M pH 7.2) for 24 h. The fixed midguts were cut with microscissors in three distinct regions: AMG1 (initial portion of the AMG), AMG2 (posterior portion of the AMG), and PMG. The samples were washed with distilled water, dehydrated in ascending ethanol series (70, 80, 90, 95 and 100%) and embedded in 2-hydroxyethyl methacrylate historesin (Leica Microsystems Heidelberg Mannhein, Germany). Serial sections of 2-μm thickness were obtained using an automatic microtome with glass knives.

The sections were subjected to different staining methods. As conventional staining methods, hematoxylin-eosin (HE) or toluidine blue staining protocols were used according to standard routine laboratory procedures. For histochemistry, the periodic acid Schiff (PAS) reaction^53^ was employed for neutral glycoproteins, neutral carbohydrates and glycogen detection; and bromophenol blue^54^ for total protein detection. After staining and drying, the slides were mounted using Eukitt (Fluka, St. Louis, MO, USA) mounting medium, analyzed, and photographed under an Olympus BX53 microscope, coupled with an Olympus DP 73 digital camera of the Laboratório de Sistemática Molecular, Departamento de Biologia Animal/UFV.

The length and width of AMG1, AMG2, and PMG of 20 insects (10 females and 10 males) were measured. The total organ length was obtained by adding the length measurements of AMG and PMG. The height of the epithelium (i.e., digestive cells), and brush border were measured separately in AMG1, AMG2, and PMG. The measurements were performed only at regular epithelia, with no cytoplasmic protrusions, and with muscular layer and brush border evident. Measurements were performed using the Image Pro-Plus 4.5 software (Media Cybernetics) in light micrographs of six females and six males.

The midgut cell organization was also analyzed in whole mounts. For this, 10 fixed and washed midguts were stained using diamidino-2-phenylindole (DAPI) (Biotium, Inc., Hayward, CA, USA) for 30 min and mounted with Mowiol (Sigma-Aldrich Brasil Ltda, São Paulo, SP, Brazil) solution. The slide preparations were photographed with a epifluorescence microscope (see above).

To examine the midgut muscle organization, 10 midguts (5 males and 5 females) were dissected and fixed for 2 h in Zamboni’s solution, rinsed thrice for 30 min in PBS/Triton X-100 1% (PBST); and incubated for 40 min in phalloidin conjugated with fluorescein isothiocyanate (FITC) (Sigma Aldrich, Sigma-Aldrich Brasil Ltda) diluted in distilled water (1:500).Then, the midguts were washed in PBS three times for 5 min, mounted on slides with Mowiol solution, and examined under a confocal laser scanning microscope (CLSM) Zeiss 510 Meta at the Núcleo de Microscopia e Microanálise (NMM) UFV.

### WGA staining

To detect glycoconjugates and polysaccharides containing β-1-4 N-acetyl-glucosamine residues, midgut (4 males and 4 females) sections of 2-μm thickness were washed in PBS two times for 10 min and incubated with for 30 min with 10 g/ml FITC-labeled lectin (WGA-FITC, Sigma Aldrich, #L4895, Israel) diluted in PBS. Sections were washed 3 times for 2 min, mounted with Mowiol solution and photographed under fluorescence microscope.

### Transmission electron microscopy (TEM)

Fragments of fixed midgut regions (AMG1, AMG2, and PMG, from 3 males and 3 females) were washed in 0.1 M cacodylate buffer pH 7.2; and post-fixed for 1 h in 1% osmium tetroxide/0.1 M cacodylate buffer pH 7.2. The samples were washed three times in distilled water and counterstained for 2 h in an aqueous solution of 3% uranyl acetate. After further washing in distilled water, samples were dehydrated in ascending ethanol series; pre-infiltrated in LRWhite resin (London Resin Company Ltd, Berkshire, England) and 100% ethanol (ethanol/resin 2:1, 1:1, and 1:2); and embedded in pure resin for subsequent polymerization at 60 °C for 48 h. Ultrathin sections (70–90 nm) were analyzed and photographed under a TEM Zeiss EM 109 at NMM/UFV.

### Scanning electron microscopy (SEM)

Whole midguts (3 males and 3 females) were fixed and post-fixed as describe above. After washing in PBS, samples were dehydrated in ascending ethanol series, critical point dried using CO_2_ and sputter coated with gold. Samples were analyzed and photographed under a SEM LEO 1430VP at NMM/UFV.

### Immunofluorescence

To identify dividing cells in the midgut, a primary antibody against the nuclear protein phospho-histone H3 (PH3) (Cell Signaling Technology, Inc., Beverly, MA, USA) was used^42^. Midguts of 10 males and 10 females were fixed in Zamboni’s fixative, washed three times for 30 min each in PBS/1% Triton X-100 (PBST) and incubated for 24 h at 4 °C in anti-PH3 primary antibody (Cell Signaling) (1:100) in 1% PBST. The samples were washed three times and incubated with a secondary antibody conjugated with FITC (Sigma) (1:500) in PBS for 24 h at 4 °C, followed by three washes in PBS of 10 min each.

For detection of cells expressing neuropeptides FMRFamide (neurons and endocrine cells), after fixation, 20 midguts (10 females and 10 males) were washed in PBST and incubated for 24 h at 4 °C with anti-FMRFamide primary antibody (Peninsula Laboratories, Inc., San Carlos, CA, USA) (1:400) in 1% PBST. Samples were washed, incubated with the secondary antibody and washed again, as described above. For both *in situ* identification of proteins (H3 and FMRF), the cell nuclei were stained with TO-PRO-3 Iodide (Life Technologies) for 30 min, washed in PBST, mounted, and analyzed under CLSM (2.2). The total number of FMRFamide-positive cells in the midgut was determined for each insect manually using the z-stack tool of multiple confocal planes.

### Controls

Midguts from six fourth instar larvae (L4) of *A. aegypti* were used as a positive control for PH3 identification^42^. As negative controls for the two proteins (H3 and FMRF) above, midguts of male and female (n = 3, each) *T. theobaldi* were treated as described in Immunofluorescence item, but without primary antibodies. As negative controls for WGA-FITC staining, histological sections of midguts of 4 individuals (2 males and 2 females) were mounted with Mowiol solution and observed under fluorescent microscope.

### Statistical analysis

Measurements of midguts and cell counts were subjected to analysis of variance (ANOVA) for variables with normal distribution, and to the Kruskal-Wallis test when non-normal distribution was found. Results were deemed significant when p < 0.05. Standard deviations (SD) were calculated using GraphPad Prism version 4.0 for Windows (GraphPad Software, San Diego, California, USA), and the data were expressed as replicate means.

## Additional Information

**How to cite this article**: Godoy, R. S. M. *et al.* Midgut of the non-hematophagous mosquito *Toxorhynchites theobaldi* (Diptera, Culicidae). *Sci. Rep.* **5**, 15836; doi: 10.1038/srep15836 (2015).

## Supplementary Material

Supplementary Information
