# Supplementary material for: Midgut of the non-hematophagous mosquito Toxorhynchites theobaldi (Diptera, Culicidae)
Source: Sci Rep. 2015 Oct 30;5:15836. doi: 10.1038/srep15836 (PMC4626790; doi:10.1038/srep15836)
Supplement: Supplementary Information [file srep15836-s1.doc]

**Midgut of the non-hematophagous mosquito *Toxorhynchites theobaldi* (Diptera, Culicidae)**

Raquel S. M. Godoy, Kenner M. Fernandes, Gustavo F. Martins*

***Corresponding author. E-mail**: [gmartins@ufv.br](mailto:gmartins@ufv.br)

**Supplementary information**

**
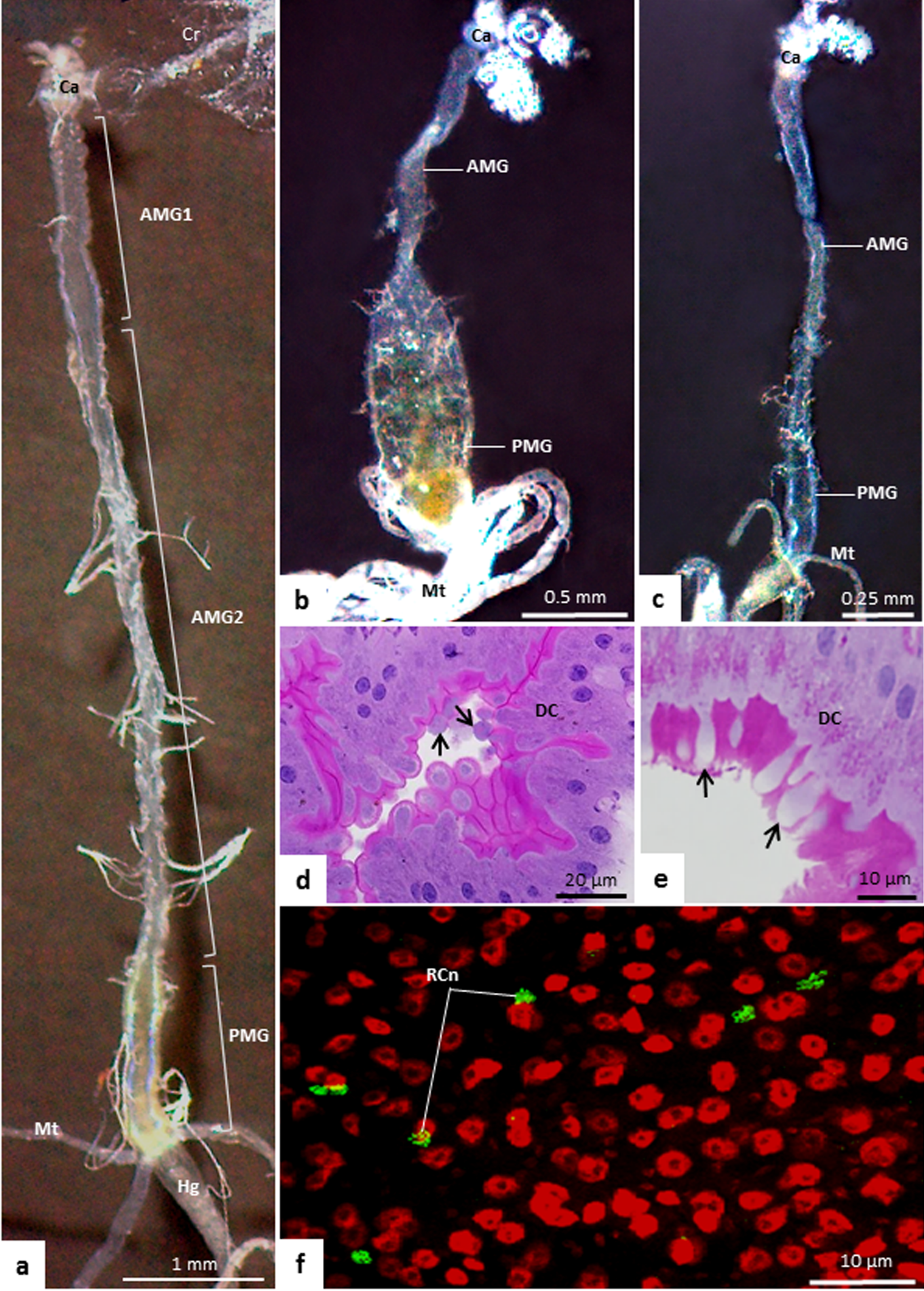
**

**Supplementary figure 1: a:** Midgut of a male *Toxorhynchites theobaldi* showing the anterior midgut (AMG) subdivided into AMG1 (short and wide) and AMG2 (long and slender), and a small and dilated posterior midgut (PMG). **b** and **c:** Unfixed midguts of adult female and male *A. aegypti* (PPCampos strain), respectively, depicting AMG and PMG. Ca: cardia; Cr: crop; Mt: Malpighian tubules; Hg: hindgut. **d** and **e:** Sections of AMG1 of female and PMG of male, respectively. Cell apexes (arrows) are negative for PAS reaction and project towards midgut lumen, resembling the process of apocrine secretion. DC: digestive cells. **f:** Nuclei of regenerative cells (RCn) positive for phospho-histone H3 (green) in the midgut of fourth larva of *A. aegypti*. Individuals were obtained from a colony of the insectary of Departamento de Biologia Geral/UFV, and were dissected and stained as described in Materials and Methods (Immunofluorescence) ^42^.
